# Optimizing neurointerventional procedures: an algorithm for embolization coil detection and automated collimation to enable dose reduction

**DOI:** 10.1117/1.JMI.11.4.044003

**Published:** 2024-07-17

**Authors:** Arpitha Ravi, Philipp Bernhardt, Mathis Hoffmann, Richard Obler, Cuong Nguyen, Andreas Berting, René Chapot, Andreas Maier

**Affiliations:** a Friedrich-Alexander-Univeristät Erlangen-Nürnberg, Pattern Recognition Lab, Department of Computer Science, Erlangen, Germany; b Siemens Healthcare GmbH, Forchheim, Germany; c Alfried Krupp Krankenhaus, Essen, Germany

**Keywords:** embolization coil, radiation dose reduction, deep-learning, blob detection, neuroradiology

## Abstract

**Purpose:**

Monitoring radiation dose and time parameters during radiological interventions is crucial, especially in neurointerventional procedures, such as aneurysm treatment with embolization coils. The algorithm presented detects the presence of these embolization coils in medical images. It establishes a bounding box as a reference for automated collimation, with the primary objective being to enhance the efficiency and safety of neurointerventional procedures by actively optimizing image quality while minimizing patient dose.

**Methods:**

Two distinct methodologies are evaluated in our study. The first involves deep learning, employing the Faster R-CNN model with a ResNet-50 FPN as a backbone and a RetinaNet model. The second method utilizes a classical blob detection approach, serving as a benchmark for comparison.

**Results:**

We performed a fivefold cross-validation, and our top-performing model achieved mean mAP@75 of 0.84 across all folds on validation data and mean mAP@75 of 0.94 on independent test data. Since we use an upscaled bounding box, achieving 100% overlap between ground truth and prediction is not necessary. To highlight the real-world applications of our algorithm, we conducted a simulation featuring a coil constructed from an alloy wire, effectively showcasing the implementation of automatic collimation. This resulted in a notable reduction in the dose area product, signifying the reduction of stochastic risks for both patients and medical staff by minimizing scatter radiation. Additionally, our algorithm assists in avoiding extreme brightness or darkness in X-ray angiography images during narrow collimation, ultimately streamlining the collimation process for physicians.

**Conclusion:**

To our knowledge, this marks the initial attempt at an approach successfully detecting embolization coils, showcasing the extended applications of integrating detection results into the X-ray angiography system. The method we present has the potential for broader application, allowing its extension to detect other medical objects utilized in interventional procedures.

## 1 Introduction

### 1.1 Radiation Dose Management in Endovascular Coil Embolization: Tackling Clinical Challenges for Safer Neuroradiology

Cerebral aneurysms pose a significant and potentially fatal health concern for millions of people worldwide. In the United States, an estimated population of 6.8 million have been diagnosed with unruptured aneurysm in the brain.^1^ These fragile blood vessel bulges require immediate medical attention to prevent the catastrophic consequences of rupture, which can lead to severe brain damage or even death.^2^ In addressing this medical challenge, endovascular coil embolization has emerged as a key intervention.

Endovascular coil embolization is a minimally invasive procedure that has brought notable advancements to the treatment of cerebral aneurysms.^3^ This method inserts platinum coils through a microcatheter to restrict blood flow to an aneurysm, reducing the risk of further enlargement or rupturing completely. Platinum coils excel in effectively sealing aneurysms, and they are also selected for their biocompatibility.^4^^,^^5^ Since they consist of platinum, their visibility in X-ray imaging is very good.

Despite advancements in neuroradiology interventions, such as endovascular coil embolization, a major challenge persists, which includes effectively managing radiation dosage and procedural time. Hence, the effective management of these factors is essential in neuroradiology due to the lengthy and time-consuming procedures involved.

To address the challenges posed by radiation exposure, collimation is employed as one of the techniques in radiological procedures.^6^^,^^7^ It enables angiographic X-ray machines to narrow the radiation field to only the necessary areas for diagnosis and therapy. This reduces the dose area product (DAP), which measures cumulative air kerma over the irradiated area and lowers radiation risks for patients and medical personnel. Additionally, it may enhance image quality^8^ by boosting the signal-to-noise ratio, reducing the scatter radiation released from the patient.

However, the current practice of collimation relies on manual adjustments of the collimator. Even though the manual process is effective, it is time-consuming and can potentially distract the operator during a procedure that requires absolute focus and precision.

Given the nature of these challenges, there is a need for methodologies that manage radiation dosage and procedural time during endovascular coil embolization. This need considers not only patient safety but also improving image quality and operational efficiency.

### 1.2 Contributions and Structure of the Paper

In our research, we propose a technique aimed at automating the detection of coils and precise alignment of collimator during neuroradiology procedures, with the goal of minimizing the (DAP) administered. In this approach, we use deep learning to train a model specifically designed for coil detection, which is then compared to a traditional blob detection method serving as a benchmark. The deep learning model outputs a bounding box that accurately pinpoints the location of the coil, serving as a reference for automatic collimation.

Our study showcases the practical application of this coil detection method, demonstrating its significance in reducing DAP. Additionally, we provide evidence supporting the claim that automatic collimation, facilitated by our deep learning model, reduces the time required for setting up collimation around the coil compared to manual methods. This contribution of DAP reduction and time efficiency illustrates its potential to enhance both safety and efficiency in neuroradiology procedures.

The paper is organized into the following sections. In Sec. 2, we explore the related work and theory on angiography systems. Section 3 outlines our approach, including dataset details, model training, and experimental setup. Section 4 demonstrates practical execution. Section 5 presents the results of our deep learning approach compared to classical blob detection and explains its application. Section 6 summarizes insights and contributions and suggests future directions.

## 2 Literature Review

### 2.1 Radiation Physics in Angiography: Strategies for Exposure Control in X-Ray Imaging

The angiography system utilizes X-ray radiation for medical imaging, offering valuable diagnostic information. However, it is essential to assess potential risks associated with prolonged exposure. These risks include deterministic effects, which pose immediate threats, and stochastic effects, which occur randomly without a specific threshold. The likelihood of stochastic risks increases proportionally with radiation exposure, and the severity of these effects is not dependent on the radiation dose. The primary stochastic effects include cancer and genetic mutations.^9^^,^^10^ This emphasizes the importance of a balanced approach in maximizing the benefits of angiography while minimizing associated risks.

DAP serves as a measurable indicator for the relative estimation of stochastic risks in interventional X-ray imaging, irrespective of anatomical factors.^11^^,^^12^ Air kerma, in units of “gray” (Gy), quantifies kinetic energy per unit mass.^13^ DAP is calculated by multiplying the patient’s exposed surface area by the air kerma. Operators and staff, while facing potential stochastic effects, are not at risk of deterministic damages as their total air kerma remains below typical thresholds.

Angiographic X-ray machines include collimators to narrow the irradiation area, reduce scatter radiation, and enhance image quality.^14^^–^^16^ Beyond collimation, careful selection of parameters by exposure control is essential. Automatic exposure rate control (AERC), widely used, monitors and halts exposure based on predetermined thresholds.^16^ AERC regulates between 3 and 5 exposure parameters and adapts to individual patient parameters, ensuring optimal exposure.^17^^,^^18^

In digital flat-panel X-ray systems, incident intensity is determined by the flat-panel detector. Although AERC is recommended for optimizing radiation exposure, challenges can arise in specific scenarios. For instance, when imaging smaller regions of interest, such as the arm or legs, where the imaged area is relatively small compared to the background, direct radiation hitting the detector can lead to premature exposure termination by the AERC. This premature halt introduces a high level of quantum noise in digital images.^16^ Another challenge occurs when imaging large metal objects. Metal objects tend to absorb more radiation than their surroundings, resulting in overexposed background areas. In such cases, the image optimization may prioritize imaging through metal objects rather than the surrounding anatomy,^16^^,^^19^ which may not be essential.

Implementing a detection algorithm can be advantageous in automatically identifying and collimating around metallic objects, thereby guiding AERC for optimal imaging outcomes. This not only enhances imaging precision but also contributes to a notable reduction in air kerma, an aspect that is elaborated in Sec. 5. The algorithm would be ideally used during the deployment of the coil. Since we use the raw data for training, it would not affect the final detection.

### 2.2 Related Work

The rise of deep learning has introduced a variety of object detection techniques, including two-stage detectors, such as Fast R-CNN and Mask R-CNN,^20^^,^^21^ as well as one-stage detectors, such as the YOLO series,^22^^–^^25^ known for their speed. The transformer,^26^ initially used in natural language processing, has emerged as a cutting-edge method for image classification.^27^ Recent advancements include nonhierarchical vision transformer architectures and ViT-YOLO,^28^ a hybrid one-stage detector. Ma et al.^29^ surveyed various object detection methods, exploring both deep learning and classical techniques for microorganism detection. Classical computer vision methods like Haar wavelet transform^30^ for face detection and blob detection^31^ for fruit detection have been significant. These blob detection methods can be differentiation-based (e.g., Laplacian of Gaussian,^32^ difference of Gaussian,^33^ and determinant-of-Hessian^33^) or thresholding-based.^34^ On the deep learning front, Bang et al.^35^ focused on AI-based collimation in fluoroscopy-guided endoscopic procedures, utilizing a secondary collimator to reduce radiation exposure. Their approach identifies activities through region of interest (ROI) detection, guiding the secondary collimator. In our project, we employ Faster R-CNN with a ResNet-50 FPN backbone and RetinaNet for coil detection, exploring its clinical benefits in neuroradiology interventions.

## 3 Methods

### 3.1 Deep Learning Network Architectures

In our experiment, we trained two distinct deep learning models: a two-stage network, Faster R-CNN,^36^ featuring a ResNet-50 FPN (feature pyramid network) as its backbone,^37^ and a one-stage network, RetinaNet. Both networks share a common feature extractor, employing an FPN with a ResNet-50 backbone.

The FPN serves as a feature extractor by taking an input image and generating feature maps at various levels in a fully convolutional manner.^37^ This approach ensures proportionally sized feature maps, enhancing the model’s ability to detect objects at different scales. The ResNet-50 architecture is utilized as the backbone for this feature extraction process.

The following sections delve into the specific details of each architecture.

#### 3.1.1 Faster-RCNN

**Fig. 1:**
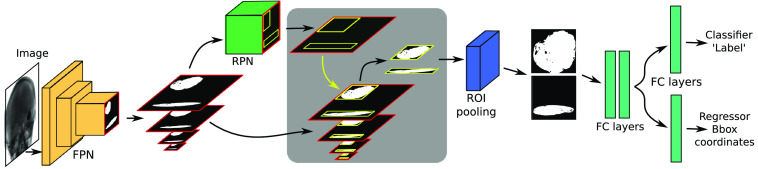
Proposed method for object detection is illustrated as follows: an input image is fed into the FPN, generating feature maps at various scales. These feature maps undergo processing in the RPN, yielding object proposals accompanied by objectness scores. The generated proposals, along with the feature maps, serve as input to the ROI pooling, where the feature maps are converted to a fixed dimension. Subsequently, these data are input into the Fast R-CNN detector, comprising two fully connected layers (FC layers) for proposal and feature map processing. The output of these FC layers is then directed through another FC layer for classification, determining the class label, and an additional FC layer for regression, obtaining the bounding box coordinates.

This model is designed for object detection tasks and operates in two stages: (a) region proposal network (RPN) based on deep convolution layers and (b) Fast R-CNN detector. The FPN is a crucial component, consisting of a bottom-up pathway and a top-down pathway that generates feature maps at various scales. The resulting feature maps from the FPN are input to the RPN, which produces rectangular object proposals with associated objectness scores.^38^ These proposals, representing single-scaled features, undergo ROI pooling and are then processed by the Fast R-CNN head to yield the final predictions and bounding boxes (for a visual representation, refer to Fig. 1).

#### 3.1.2 RetinaNet

**Fig. 2:**
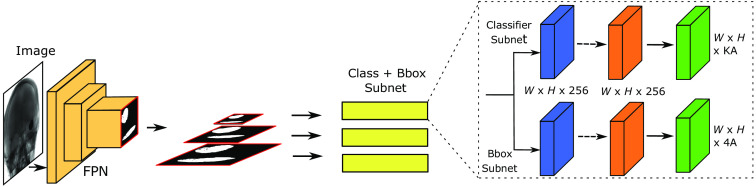
Proposed method for object detection is illustrated as follows: the input image is fed into the FPN, which generates feature maps in different scales, similar to Faster-RCNN. The class + bounding box subnet is attached to every image output from FPN, one for classifying the anchor boxes and the other for regressing from anchor boxes to the object bounding box. The class subnetwork, located in the top layer generates a class label, while the bottom layer’s bounding box regression subnetwork generates the bounding box coordinates.^39^

RetinaNet, a one-stage model, shares its feature extractor with the Faster R-CNN model. However, RetinaNet enhances this feature extractor by incorporating two subnetworks: one for classifying anchor boxes and another for regressing from anchor boxes to actual object boxes, thereby functioning as a single-stage detector. The design aims to streamline the network for faster detection without compromising accuracy. The RetinaNet model adopts the focal loss function. This dynamic scaling cross-entropy loss incorporates a scaling factor that diminishes to zero as confidence in the correct class increases^39^ (for a visual representation, refer to Fig. 2).

### 3.2 Blob Detection

**Fig. 3:**
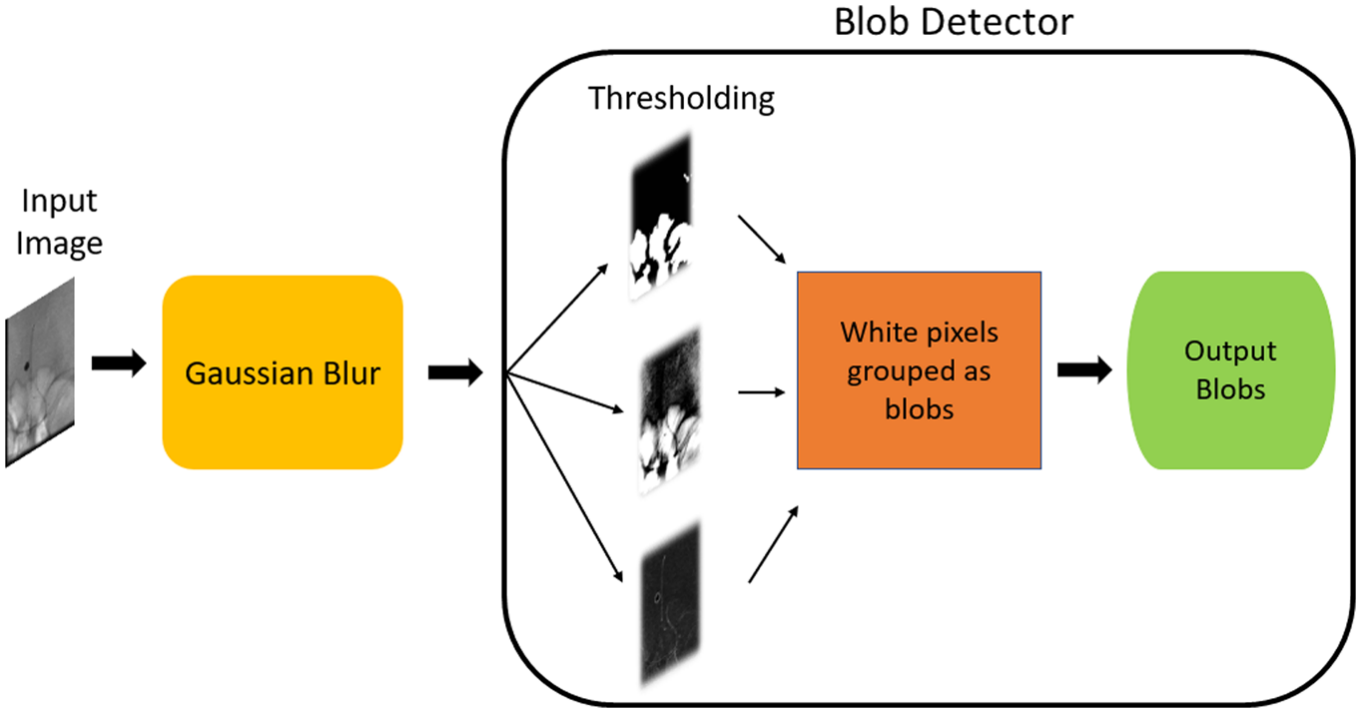
Illustration of the proposed methodology of the Blob detection.

In our dataset, the coils are represented as blobs, where “blobs” refer to clusters of interconnected pixels sharing similar characteristics. To benchmark the performance against a deep learning approach, we evaluate a classical blob detection method on the independent test data. This method employs a thresholding-based technique from the cv2 module in Python.^34^ Figure 3 illustrates the schematic representation of the blob detector used in this paper.

The steps involved in the proposed methodology of the blob detection are as follows.

• The input image is blurred using Gaussian blur to enhance the variance and diminish local peaks.
• The blurred input images are passed through the blob detector, where they undergo thresholding, resulting in multiple binary images.
• White pixels in each binary image are grouped into blobs.
• Centers for these binary blobs are computed, and a minimum distance threshold is set. If the distance between any two blobs is below this minimum, the blobs are merged.
• The centers, along with the radii, are then calculated and returned for the newly merged blobs.

The content presented here is entirely self-authored; however, it has undergone paraphrasing with the assistance of ChatGPT to express the information in varied wording.

## 4 Experiments

This section provides comprehensive insights into data acquisition and preparation, offering a detailed examination of the experimental setup. This encompasses the architecture of both the deep learning network and blob detection, along with the evaluation metrics employed. Furthermore, we provide the details of the specific configuration utilized to showcase the application of the coil detection method.

### 4.1 Data Characteristics

The dataset consists of radiographs obtained during neuroradiology interventions, comprising sequences with varying numbers of frames. The dataset comprises patient data with varying numbers of sequences per patient, depending on whether the procedure is diagnostic or interventional. Diagnostic procedures contain fewer sequences as the procedures are conducted to examine potential vascular issues or confirm intervention success at a later date. In contrast, interventional procedures result in a larger number of acquired sequences, due to increased complexity and ultimately longer examination times. For training purposes, only one frame containing the coil(s) from each sequence was considered. The dataset includes images consisting of varying numbers of coils with different shapes and sizes. The database also includes images of coils acquired in the presence of other medical devices like catheters, guidewires, and so on.

**Fig. 4:**
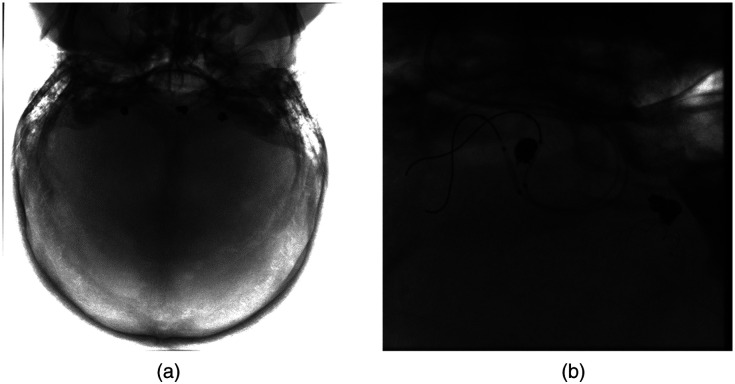
Example coil images used in the study: (a) example image representing three coils of different shapes and (b) example image representing coils along with catheters and guidewires.

Figure 4 presents example coil images used in this study. The images are used in a so-called raw format. Raw data in this context refer to images directly obtained from the X-ray angiography system without undergoing any preprocessing or filtering. The images are only corrected for detector artifacts. The data also do not contain any patient information. Since the project aims at real-time detection, raw data were used.

**Fig. 5:**
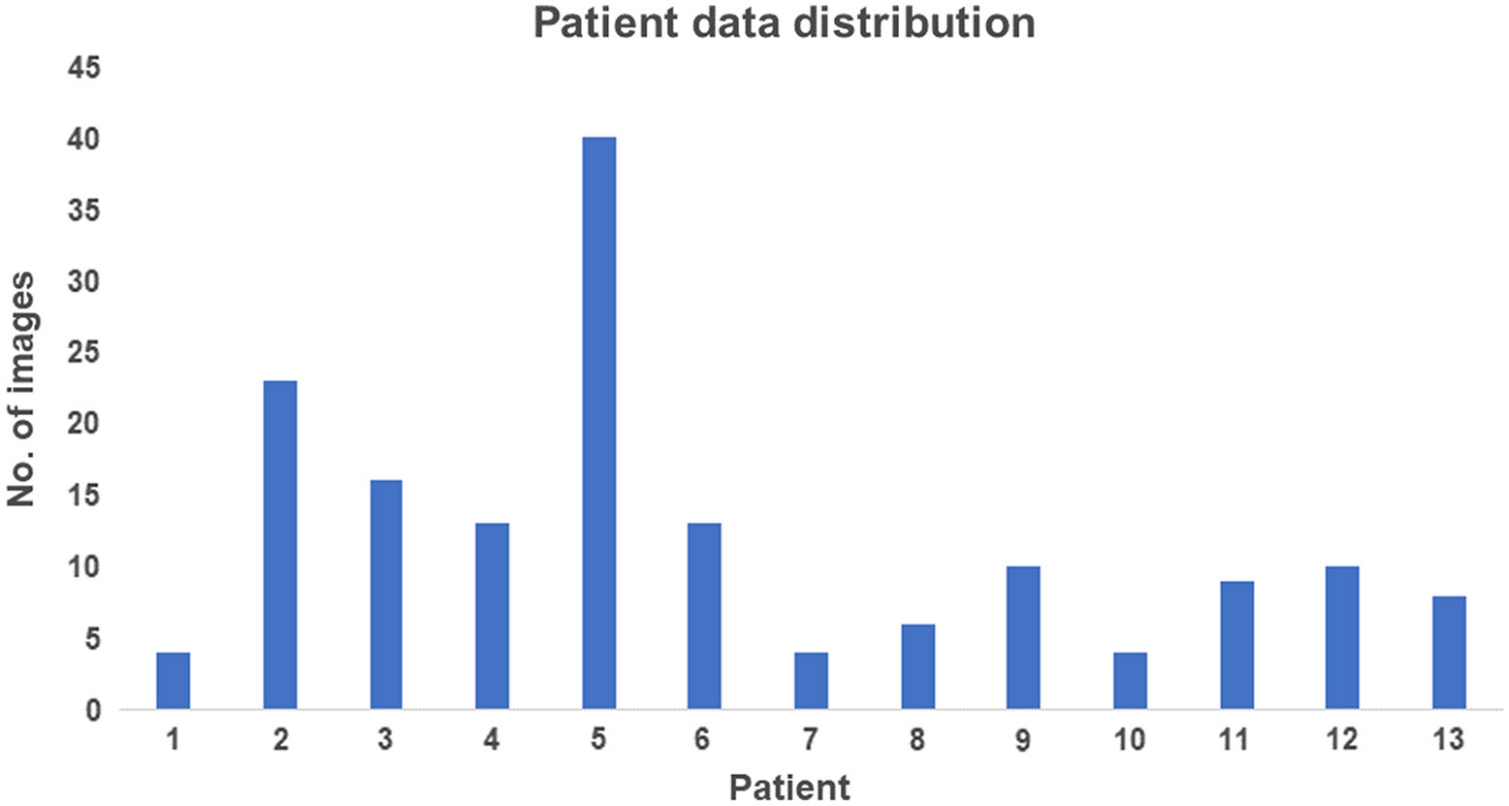
Plot depicting patient distribution. The x axis represents the patient number and the y axis represents the number of images corresponding to that patient.

Given the variability in imaging and acquisition parameters among surgeons and patient demographics, we employed a multisite dataset consisting of 160 images from 13 patients obtained from Siemens X-ray biplane angiography machines. Hence, every coil image used provided two different projections of the same coil. The dataset encompasses images from both diagnostic and interventional procedures. However, there is an imbalance in patient distribution, with fewer images from patients undergoing diagnostic procedures compared to those undergoing an intervention, as illustrated in Fig. 5. The images also included coils with different zoom formats and levels of collimation, various acquisition phases in the procedure.

#### 4.1.1 Data preparation and labeling

Given that the images are in raw format, adjustments are made to correct for flip, and rotation, and the collimator area is cropped. The collimator cropping parameters necessitate the images to be initially corrected for flipping and rotation before applying them. Despite the collimator being a black region with nondiagnostic information, its presence adds noise during training. To mitigate potential adverse effects during neural network optimization, the collimator region was removed using specific parameters obtained and stored during acquisitions.^40^ The collimator-cropped images underwent normalization using the standard scaler technique (zero mean unit variance)^41^ and were subsequently saved as TIFF files. These files were then utilized for labeling purposes using the labelImg tool^42^ and for training purposes in the same format. The annotation and evaluation of this data are based on the Pascal VOC metrics.^43^ The images were self-annotated under the supervision of clinical experts with more than 10 years of experience to ensure accuracy and reliability in object identification and the annotated coils were assigned to coil class. For faster training, original variable-resolution images were resized to a fixed resolution of 512×512 before input to the deep learning network.

#### 4.1.2 Data distribution

To ensure robust evaluation, a fivefold cross-validation approach was employed. To account for the imbalance in the patient distribution, we split the data into five folds such that every patient has been part of the validation data at least in one of the five folds to avoid any bias. The remaining data are used for training. This split is also performed patient wise so for every fold there are either two or three patients in the validation set and the rest are in training. The data distribution between training, and validation sets is presented in Table. 1. We also present the patient number involved in every fold of validation data.

**Table 1:** Number of images distributed between the training and validation data in all the five folds. It also presents the patient number utilized for each fold in validation data.

Fold	Training	Validation	Patient number in validation data
1	128	32	9,12,1,13
2	131	29	2,8
3	131	29	3,4
4	130	30	6,11,10,7
5	120	40	5

Additionally, an independent test set, sourced from a single site, featuring 12 images that included coils of different shapes and sizes was used to evaluate the best-performing model.

### 4.2 Training and Validation Protocol

In this section, we delve into the training process of our deep learning model and the working of the blob detector. We discuss the data inputs utilized and the training methodology, including hyperparameter settings.

#### 4.2.1 Faster-RCNN

The data distribution used for the model is described in Sec. 4.1.2. The normalized TIFF images and the bounding box annotations are provided as input to the pretrained Faster-RCNN model for training. The output of this model is bounding box coordinates for the detected object and the corresponding label which is the detected coil in our context.

To enhance the performance of the model, we also use data augmentation. The techniques used are flip and random rotation of 90 deg, Gaussian blurring, and pixel dropout. The data augmentation is performed only on the training data. A batch size of 2 was used due to the image’s high resolution and limited memory capability. Due to this, the learning rate used was 0.0001, and the optimizer used was Adam. The training was performed on a Quadro RTX 6000 GPU.

#### 4.2.2 RetinaNet

The model’s data distribution is explained in Sec. 4.1.2. The normalized TIFF images and the bounding box annotations are provided as input to the RetinaNet model for training. The output of this model is bounding box coordinates for the detected object and the corresponding label which is the coil. We use the same data augmentation techniques and hyperparameters as described for the Faster-RCNN model.

### 4.3 Blob Detection

The overall configuration of the blob detector is detailed in Sec. 3.2. Figure 3 illustrates the schematic representation of the blob detector.

Similar to the deep learning models, the input images are used in a TIFF format. We use the images from the independent test set as input to the blob detector. Before subjecting the images to the blob detector, they undergo blurring to amplify variance and diminish local peaks, enhancing subsequent thresholding. Gaussian blurring with a sigma value of 9 is applied to achieve this. The blurred image is provided as input to the blob detector. The output is a thresholded image featuring detected blob key points represented by red circles. Manual tuning of several parameters is possible for blob detection, but for simplicity, only the blob area is utilized as a parameter in this study. The area of the largest and smallest coils serves as the maximum and minimum thresholds, respectively, ensuring that blobs outside these thresholds are disregarded. Also, manual tuning makes the blob detector less robust and increases complexity. The output of the blob detector is a set of key points represented by a circle around the detected blob. In our evaluation process, we augment this representation by drawing a bounding box around the identified circle. This bounding box serves as the predicted region, which is subsequently compared with the ground truth box to compute the intersection over union (IoU) for performance assessment.

### 4.4 Applications

To demonstrate the applications of the proposed method, we performed experiments, which are discussed in the following sections.

#### 4.4.1 Experiment 1

The experiment aimed to simulate coil detection and automatic collimation around it by utilizing bounding box coordinates as a reference. We also want to demonstrate the comparison of using manual collimation against automatic collimation. To emulate the coil, a thin wire composed of 95% tin, 3.8% silver, and 1.2% copper was twisted and crumbled to achieve a coil-like shape. The coil was positioned at different regions of a skull phantom for imaging purposes, including the top and bottom of the phantom to test extreme cases.

We performed five image acquisitions with the simulated coil using different levels of manual collimation. Subsequently, the acquired images were processed through our Faster-RCNN coil detection algorithm to generate the bounding box coordinates. An upscaled version of the bounding box was employed for collimation, allowing for enhanced visualization of the coil’s surroundings. The degree of scaling was determined by an expert for experimental purposes. The bounding box coordinates obtained from our coil detector were then used for collimation, and a subsequent image was acquired.

DAP values were recorded for both manual and automatic collimation for comparative analysis. The experiment was performed on the Artis Pheno X-ray angiography system with a detector size 30×40, at the lab facility in Siemens Healthineers.

#### 4.4.2 Experiment 2

To demonstrate the adaptability of the coil detection method across different scenarios, an experiment was conducted to evaluate its performance in certain uncommon situations. It includes instances where the coils are larger or tightly collimated, where the coil dominates the surroundings and the system starts to optimize the exposure on passing through the coil making the surroundings very bright with no visible information. We quantify the reference point air kerma and DAP savings by computing these values with and without the system’s awareness of the coil’s presence. Thereby demonstrating the application of the coil detection method. The experiment involved using a skull phantom, with a coin placed on the head to simulate a coil. The imaging process started with capturing an image of the entire skull, noting the initial reference point air kerma and DAP values. Following this, two additional measurements were taken after closely collimating around the coin. Since the reference point air kerma and DAP measurements are relative, the initial values were recorded before releasing the X-rays, and the final values were noted after the exposure. The absolute values were obtained by subtracting the final from the initial values.

The second measurement was conducted with the system being unaware of the object in the image, while the final measurement was made after making the system aware of the object, as would be the case when utilizing our detection algorithm. The experiment was performed on the Artis Icono X-ray angiography system with a detector size 30×40, at the lab facility in Siemens Healthineers.

#### 4.4.3 Experiment 3

The purpose of this experiment was to determine the average time required for manual collimation. A setup similar to experiment 2 was arranged for this assessment. Five clinical experts participated in the experiment. The coin, utilized as a reference for the coil, was imaged, and the time taken for manual collimation-recorded from the initiation to the completion of the process, was documented.

To introduce variability, the machine was configured to position the coin randomly within the field of view. The experts were tasked with aligning the C-arm to the center of the coin and subsequently collimating around it. Each expert performed three trials, and the average time across these trials was computed for analysis. The experiment was conducted with two different systems, Artis Icono 30×40  cm detector and Artis Icono 21×21  cm detector.

### 4.5 Evaluation Metrics

For the evaluation of the deep learning model, we use mean average precision (mAP) as the metric and IOU for evaluating the blob detection model.

#### 4.5.1 Intersection over union

IOU is a metric most commonly used in object detection, segmentation, and tracking tasks. It is based on the overlap between ground truth and the predicted bounding box. It is the ratio of the area of overlap and the area of union between the ground truth and prediction. It is also known as the Jaccard index:

$$\mathrm{IOU}=\frac{\left|A\cap B\right|}{\left|A\cup B\right|}=\frac{\left|I\right|}{\left|U\right|}.$$ (1)

In Eq. (1), A and B refer to the ground truth and predicted bounding boxes, respectively. The IOU value ranges between 0 and 1. If there is no overlap, then the IOU is 0 and if there is complete overlap, IOU is 1.^44^^,^^45^

#### 4.5.2 Mean average precision

It is a metric generally used for the evaluation of object detection tasks. For calculating mAP, the average precision for each class is computed, and the average over all the classes is considered.^46^ The formula for mAP is as follows:

$$\mathrm{mAP}=\frac{1}{N}\overset{N}{\underset{i=1}{\sum }}{\mathrm{AP}}_{i},$$ (2)

where N refers to the total number of classes and AP refers to the average precision. The AP calculation is based on IoU thresholds. These AP values differ for different thresholds. To avoid the uncertainty in picking an optimal IoU threshold, we calculate the AP over different thresholds.^46^ The average is taken over all classes and all the samples for the different thresholds. The mAP values range between 0 and 1, with 0 being the least and 1 being the best mAP score.

#### 4.5.3 Statiscal significance test

In our results, we want to perform a comparison of independent measurements/results to determine the significant difference between them. For this calculation, we utilized the Mann–Whitney U test^47^ implemented with the “mannwhitneyu” function from the Python scipy library.^48^ This method provided two key outputs: the Mann–Whitney U statistic and the associated p-value (probability value). Subsequently, we compared the obtained p-value against a predetermined significance level of 0.05 to determine the acceptance or rejection of the null hypothesis.

## 5 Results and Discussion

This segment is divided into two sections. The first section includes evaluating the deep learning models for object detection and comparing the best deep learning model with a classical blob detection method. The second section focuses on the results obtained from the application of the method for automatic collimation and the resulting reference point air kerma and time reduction.

### 5.1 Deep Learning Model Results

First, we assess both deep learning models using the mAP@75 metric and subsequently subject the best-performing model to further testing on independent test data.

**Fig. 6:**
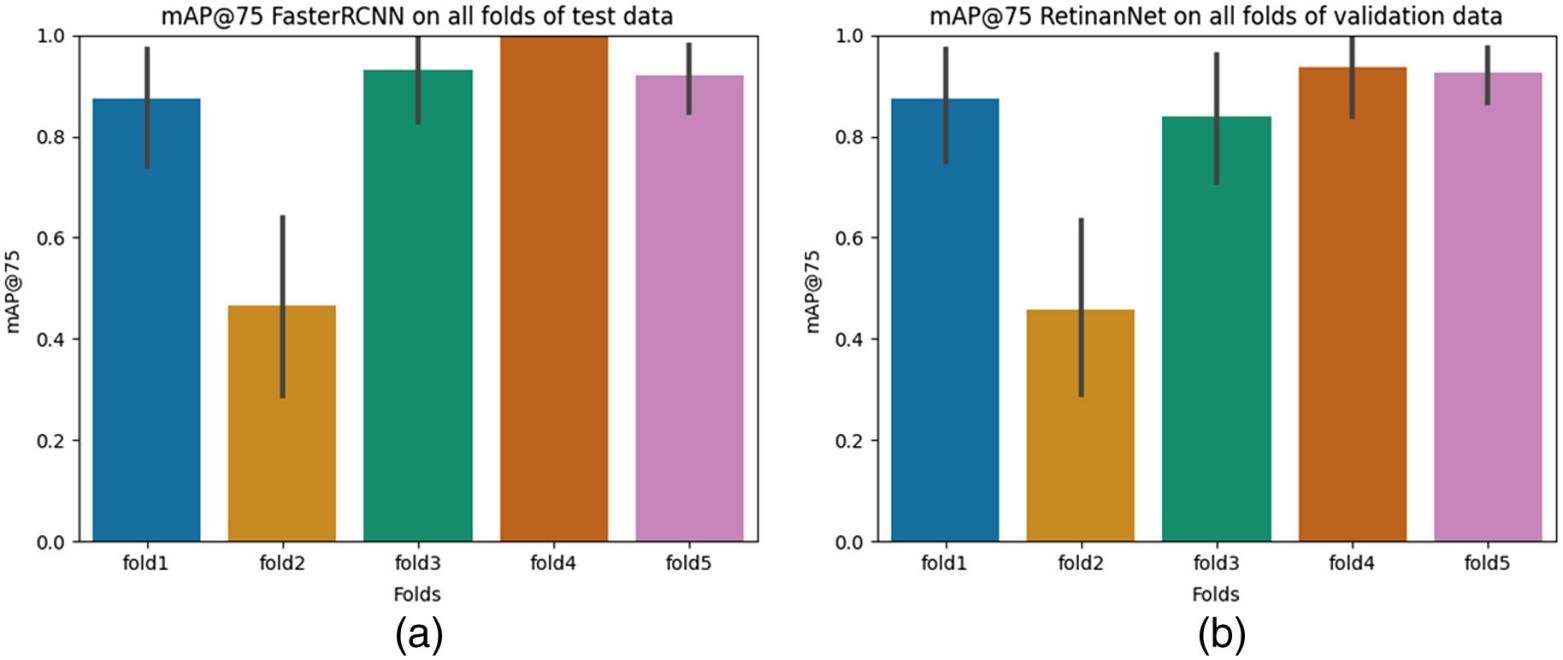
Quantitative evaluation results of both deep learning models. (a) Plot presenting mean mAP@75 obtained by Faster-RCNN model on validation data of individual folds. (b) Plot depicting mean mAP@75 obtained by RetinaNet model on validation data of individual folds.

**Fig. 7:**
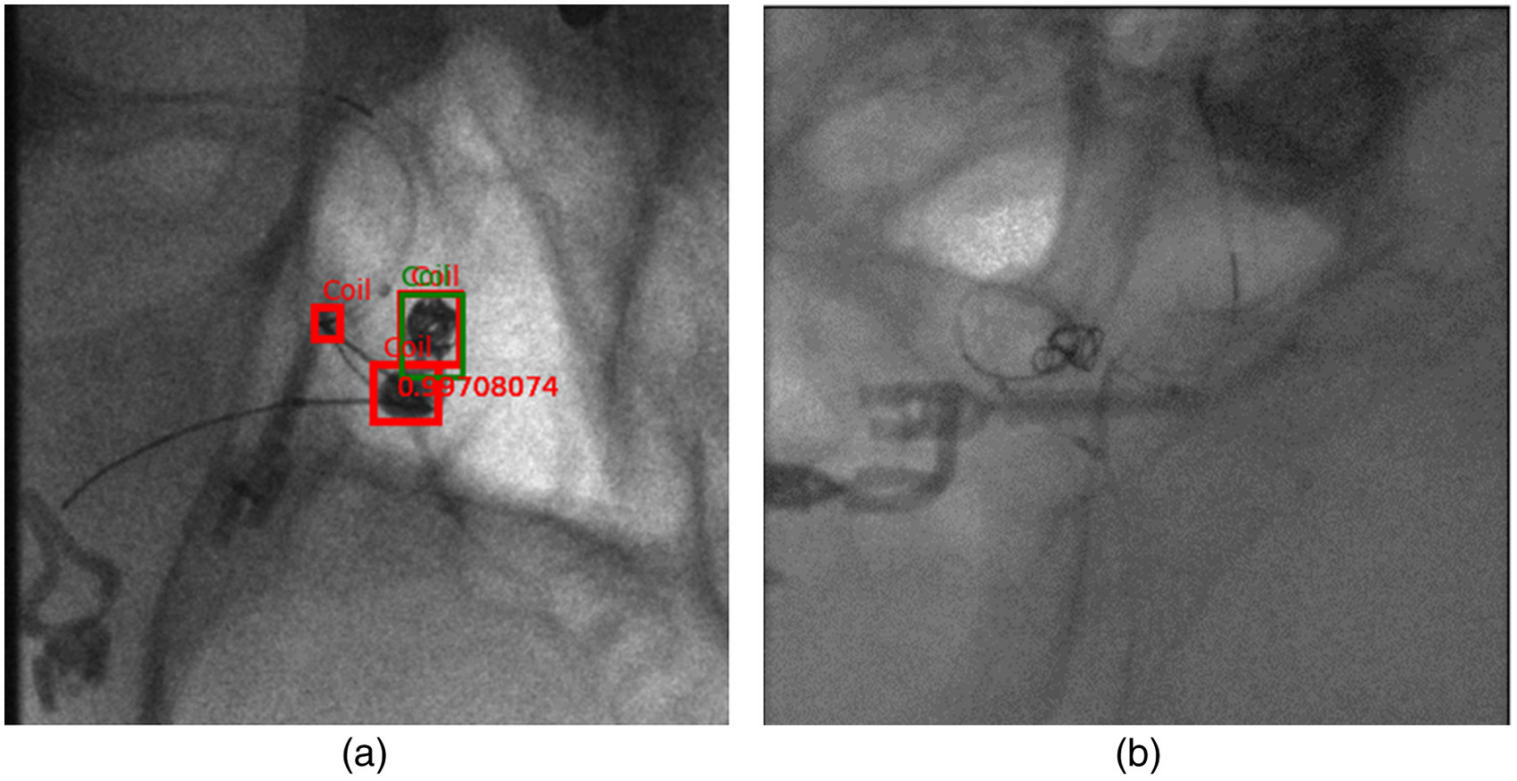
Example figures from fold 2 of the validation data. (a) A prediction image from the Faster-RCNN model that has many false positive predictions. (b) Image that includes only a framing coil in the presence of other devices.

Figure 6 illustrates the mean mAP@75 scores across all five folds of validation data, and Table 2 represents the standard deviation for all five folds of validation data for both deep learning models. Each bar represents the mAP@75 for that particular fold’s validation data. The mAP@75 metric, determined at a 75% IOU threshold, is essential in this study, aiming to identify coils and utilize bounding boxes as references for collimation. Since we only use an upscaled version of the bounding box, an exact 100% overlap between ground truth and prediction is not imperative. Therefore, we consider an IOU threshold of 75 as a suitable metric for our objectives. We observed a notable decrease in the mAP@75 obtained by both models on fold 2 validation data compared to other folds. This decrease is primarily attributed to the inclusion of data from patient 2 in the fold, which contains challenging examples not present in the training data. Example images from the fold 2 validation data are illustrated in Fig. 7. It consists of images from a patient who underwent a craniotomy for aneurysm treatment, which is not part of the training data. Figure 7(a) displays a prediction image generated by the Faster R-CNN model, revealing the presence of other devices falsely identified as coils alongside correct coil predictions. Figure 7(b) presents an image containing only a framing coil (first coil deployed during the coiling process, which makes it difficult for the network to generalize on them as it is not part of the training data. There was no prediction by the model on this image. Consequently, these images contribute to an overall decrease in mAP@75 scores. Therefore, we also report overall scores excluding fold 2, resulting in mAP@75 values of 0.93 for the Faster R-CNN model and 0.9 for the RetinaNet model.

**Table 2:** Standard deviation values of Faster-RCNN and RetinaNet models on validation data.

Model	Standard deviation
Faster-RCNN	±0.34
	±0.5
	±0.26
	±0
	±0.22
RetinaNet	±0.34
	±0.48
	±0.36
	±0.22
	±0.17

**Fig. 8:**
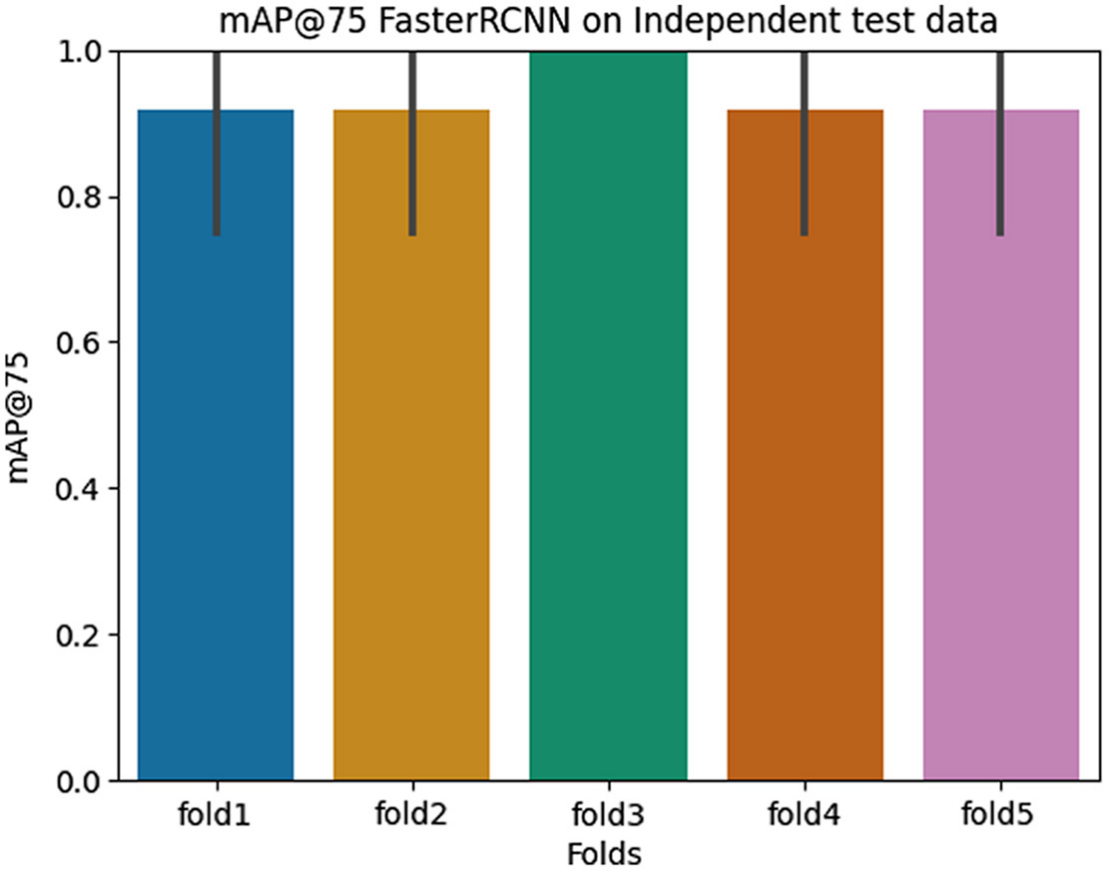
Plot presenting the mean mAP@75 obtained from the best performing model (Faster-RCNN) on the independent test data.

To assess the difference between both models statistically, we perform the Mann–Whitney U test as described in Sec. 4.5.3, which resulted in a Mann–Whitney U statistic of 15 and a p-value of 0.67, indicating no statistically significant difference between them. However, the mean mAP@75 across all folds for the RetinaNet model is 0.81 and the Faster-RCNN model is 0.84, which shows a slightly better performance of the model. Hence, we use this model for further assessment of the independent test data. Figure 8 presents the mAP@75 scores obtained by all the folds of the Faster-RCNN model on the independent test data.

Our results demonstrate the Faster-RCNN model’s ability to generalize well across various image types in both the test and independent test datasets. Specifically, we achieved a mean mAP@75 of 0.84 over all the folds on the validation data and a mean mAP@75 of 0.94 on the independent test data using the Faster-RCNN model.

**Fig. 9:**
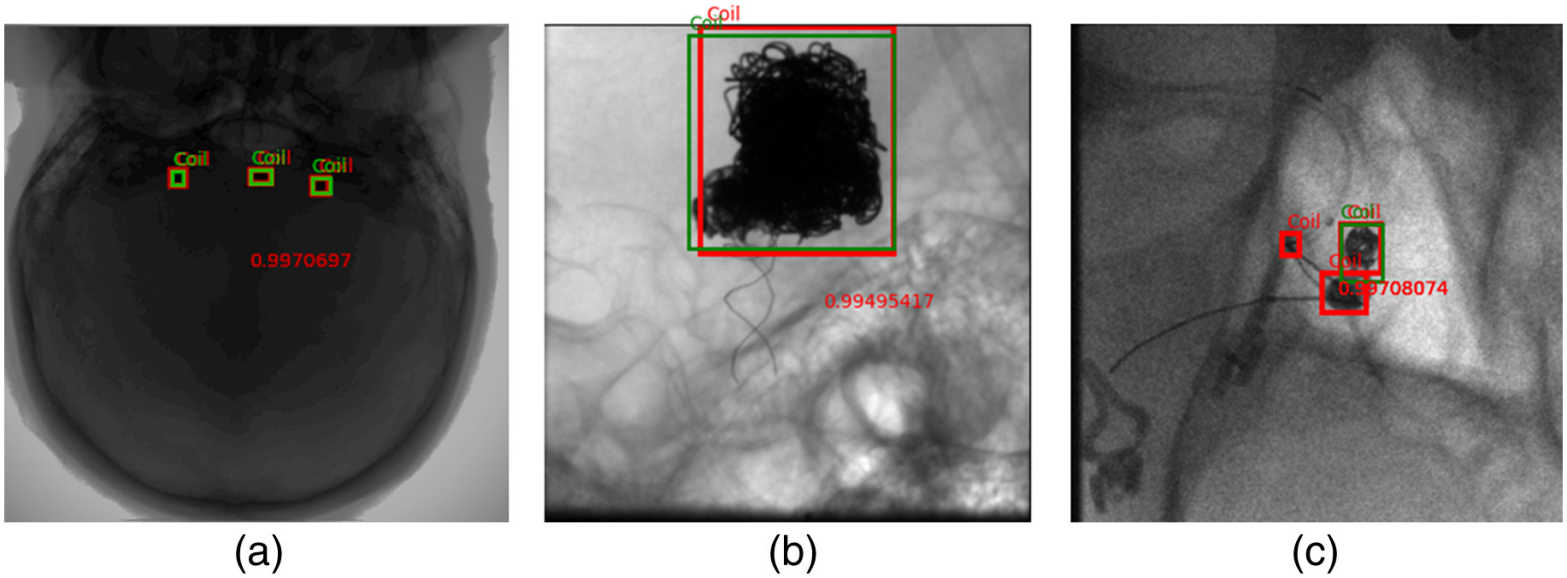
Faster-RCNN model predictions on validation and independent test data. The green bounding box indicates the ground truth and the red box is the prediction of the model with the confidence score printed in red: deep learning model prediction on an image with (a) many coils and improperly windowed and (b) large and irregularly shaped coil. (c) Example image showing the false positive detection along with the original coil.

In Fig. 9, we present the qualitative outcomes of the Faster-RCNN model. It is evident that the model demonstrates robust generalization, yielding high confidence scores across images with varying coil sizes and quantities. Figure 9(a) highlights the model’s ability to detect even small coils with notable confidence, regardless of the total number of coils present. Notably, the network exhibits the capability to predict coils in improperly windowed images with suboptimal contrast for visual perception. This is crucial because the raw images are not necessarily perfectly windowed in live imaging. The original image properly windowed is presented in Fig. 4(a).

Figure 9(b) further emphasizes the model’s reliability, showcasing accurate predictions even in the case of large, irregularly shaped coils. Despite these successes, certain false positive instances are evident, as depicted in Fig. 9(c). The network misclassifies areas with other medical devices as parts of embolization coils. This misclassification is likely due to the shared features between these objects and coils and also the absence of such samples in training data, posing a challenge to the network’s discrimination. The limited number of such samples in the training data contributes to this challenge. We are confident that augmenting the training data with a more comprehensive set of such samples would improve the network’s ability to avoid such cases.

### 5.2 Evaluation of Blob Detection Model

In this section, we assess the blob detection method’s results, aiming to compare its performance with the Faster-RCNN. The evaluation employs the IoU metric instead of mAP due to the absence of prediction scores in the blob detection model.

Table 3 presents the mean IoU and standard deviation across independent test data, excluding images without coils for both deep learning and blob detection methods. The results show lower mean IoU scores for the blob detection method compared to the Faster-RCNN model, indicating challenges in generalizing across various coil shapes when using blob detection.

**Table 3:** Comparison of evaluation of the blob detection method and Faster-RCNN method on independent test data presenting mean IoU and standard deviation.

Method	No. of samples	mean IoU	Standard deviation
Blob detection	12	0.41	0.4
Faster-RCNN	12	0.87	0.029

**Fig. 10:**
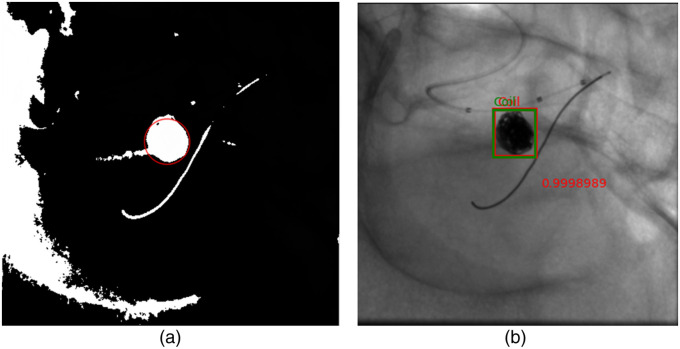
Comparison of the predictions provided by Faster-RCNN model compared to thresholding followed by blob detection: (a) Blob detection result on the image with a circular coil and (b) Faster-RCNN model prediction of the same image.

**Fig. 11:**
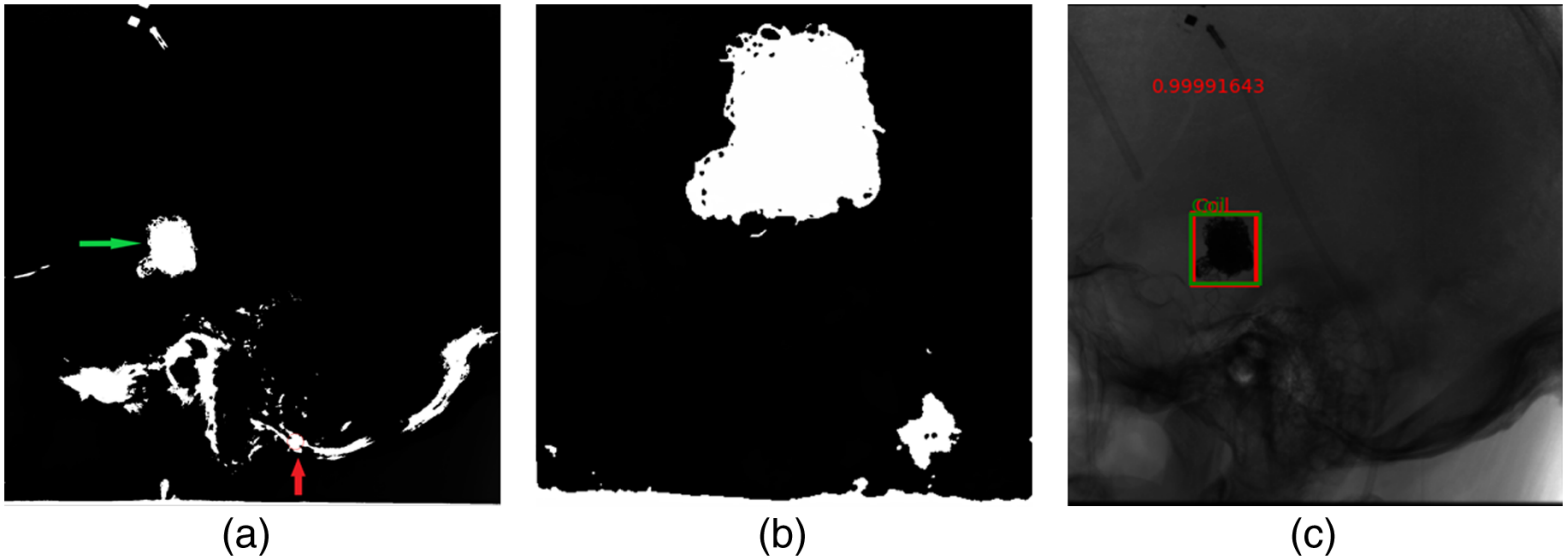
Resulting thresholding images along with keypoints presented in red circles, after passing the images through the blob detection algorithm: (a) Blob detection results on the image with an irregularly shaped coil; (b) Blob detection results on an image with large and irregularly shaped coil; and (c) Faster-RCNN model result of the same image as (a).

Figure 10 illustrates a comparison of results between the Faster-RCNN model and the blob detector on an image with a circular coil, showcasing clear detection by both methods. However, when applied to an image with an irregularly shaped coil [Fig. 11(a)], the blob detector produces a false positive, which is indicated by the red arrow and the green arrow indicates the coil. However, the Faster-RCNN model accurately identifies the coil as shown in Fig. 11(c).

Figures 11(b) and 9(b) display results for a large irregular coil, revealing the blob detector’s failure to detect it, despite setting the threshold appropriately. The Faster-RCNN model exhibits greater robustness, with average IoU scores over twice as high as the blob detector. A possible explanation for this disparity lies in the blob detector results, where other areas in the output images are highlighted for the same threshold as shown in Fig. 11(a) even though we blurred the image before providing it as input to the algorithm to prevent local peaks. This occurs even when the coil seems to be the darkest region, leading to false positives.

Despite the possibility of potential improvements for the blob detector, parameter tuning for each coil type is time-consuming. Moreover, the blob detection method is constrained by its inability to handle improperly windowed images, such as Fig. 9(a). The method’s output to such data is just a black image with no detections, a limitation not present in the Faster-RCNN model.

### 5.3 Application Results

This section presents the results of the applications derived using the Faster-RCNN model and its clinical benefits.

#### 5.3.1 Results: experiment 1

**Fig. 12:**
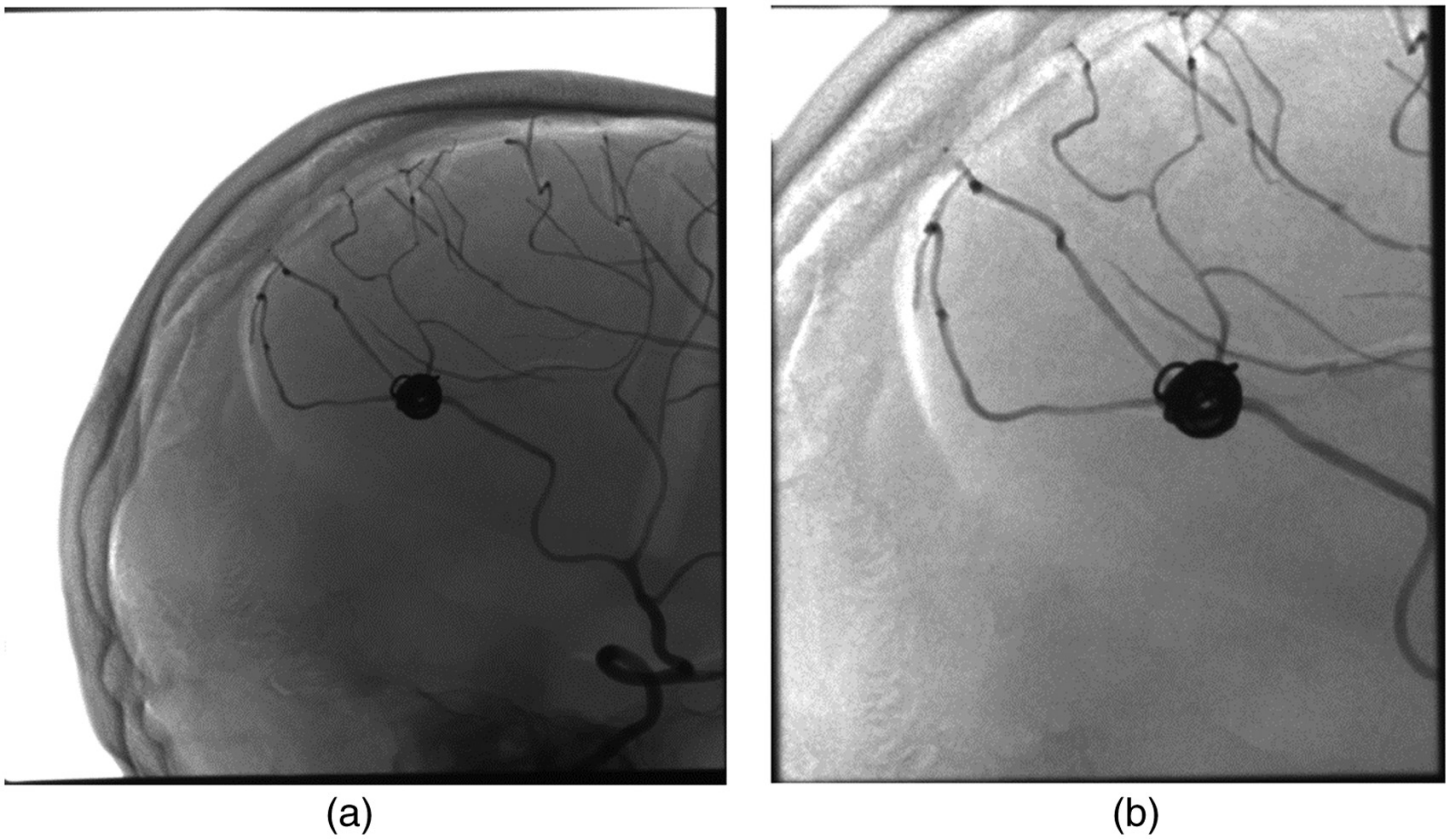
Radiographs obtained using the simulated coil, with manual and auto collimation: (a) Partially collimated image obtained using the simulated coil. This image is further used on our object detection algorithm for detecting the coil. (b) Image acquisition was performed after collimation around coil using the bounding box coordinates generated by our object detection method (Faster-RCNN).

An example radiograph captured during experiment 1 is illustrated in Fig. 12. Figure 12(a) displays a partially and manually collimated head image, serving as the input to the Faster-RCNN detection method to identify the coil. The resulting bounding box coordinates, postscaling, serve as the reference values for subsequent collimation. The scaling in this case is performed manually in the coil detection program by adding a scaling factor to the obtained bounding box coordinates. However, as explained, this scaling factor is only for experimental purposes. This process leads to the generation of the collimated image depicted in Fig. 12(b). We repeat this experiment with different levels of manual collimation. It is repeated five times to obtain the DAP values between manual and automatic collimation. Table 4 provides the DAP values between manual and automatic collimation. We statistically assess the difference between the DAP values using Mann–Whitney U test as described in Sec. 4.5.3. It resulted in a Mann–Whitney U statistic of 25 and p-value of 0.01. The Mann–Whitney U statistic of 25 indicates that one sample’s ranks are typically higher than the other, suggesting a difference in their distributions. Since the p-value of 0.01 is below the significance level, we conclude that there is a significant difference in DAP between manual and automated collimation. This validates the effectiveness of our Faster-RCNN model in reducing DAP. Furthermore, when we used the manual collimation images on the blob detection method, it did not yield any detected coils due to the windowing problem.

**Table 4:** DAP measurements in ($\mu {\mathrm{Gym}}^{2}$) obtained with five different collimation settings, (1) manual includes different levels of manual collimation including some full head acquisitions and (2) automatic includes processing the manually collimated images through Faster-RCNN to obtain bounding box coordinates and collimating using these coordinates as reference.

Experiment	Manual	Automatic
1	290.78	36.09
2	134.27	31.78
3	250.33	68.61
4	180.77	31.78
5	132.61	45.01

#### 5.3.2 Results: experiment 2

**Fig. 13:**
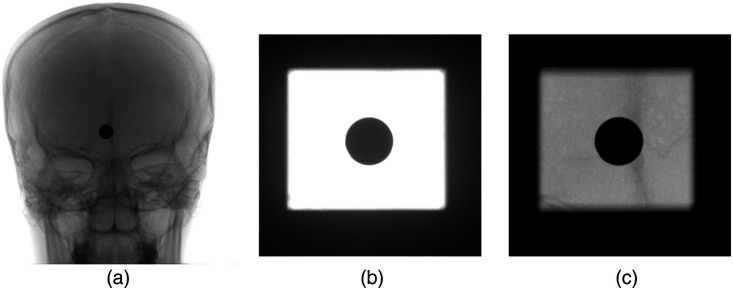
Resulting radiographs obtained by performing measurements with different settings of the X-ray angiography system. (a) Full head image acquisition with coil and without collimation. Measurement performed with collimation around coil but the system is (b) unaware of the presence of coil and (c) aware of the presence of coil. These acquisitions were performed to demonstrate reference point air kerma reduction and better visualization.

Table 5 displays the recorded reference point air kerma and DAP values following the completion of experiment 2. The initial row in Table 5 corresponds to the outcomes of the first measurement conducted without collimation, wherein the full head was imaged without specific object focus. It is evident that the reference point air kerma value is relatively low (27 mGy); however, the DAP is notably high at 526.2 ($\mu {\mathrm{Gym}}^{2}$). This discrepancy arises from the larger coverage area, as depicted in Fig. 13(a), in contrast to the collimated regions illustrated in Figs. 13(b) and 13(c).

**Table 5:** Reference point air kerma and DAP measurements obtained with different settings, (1) full head image acquisition, (2) system unaware of the presence of coil, and (3) system aware of the presence of coil depicting reduced reference point air kerma and DAP compared to the other two settings (highlighted in bold).

Collimation dose	Reference point air kerma in (mGy)	DAP in ($\mu {\mathrm{Gym}}^{2}$)
Full head	27	526.2
Coil unknown	336	166.34
Coil known	**25**	**12.14**

The following two measurements in the experiment reveal outcomes after collimating around the target coil. In the initial collimated measurement, the system lacks awareness of the coil within the image. Notably, the reference point air kerma increases to 336 mGy, a relatively high value, while the DAP decreases to 166.34 ($\mu {\mathrm{Gym}}^{2}$), which is lower than the first measurement. This discrepancy arises because the AERC in the X-ray system is unaware of the high-absorbing coil. The AERC interprets the collimated coil region as a projection of an overweight patient, optimizing imaging based on the coil (metal) rather than the surrounding area. This results in an elevated tube voltage and a higher reference point air kerma value. Additionally, the high contrast between the coil and the background makes visualizing the surroundings challenging, as depicted in Fig. 13(b). Due to this imaging optimization, the reduction in DAP does not align with the reduced area.

The current workaround involves a specific workflow where the user shifts the collimated area to a nearby soft tissue region, allowing the AERC to regulate the reference point air kerma without considering the high-absorbing coil in the image. The AERC is frozen using a process called regulation stop, after which the physician proceeds with the procedure. However, this solution is intricate, time-consuming, and does not guarantee optimal image quality and dose regulation.

To address this issue, we propose using our coil detection algorithm to inform the X-ray system about the presence of the coil. This approach enables the measurement field for reference point air kerma regulation to ignore the coil region, regulating the reference point air kerma based on the surrounding area. Consequently, the AERC no longer focuses on the coil, preventing an increase in tube voltage and resulting in a reduction of both reference point air kerma and DAP. In the third row of Table 5, the reference point air kerma decreases from 336 to 25 mGy in a subsequent measurement where the system is made aware of the coil presence, resulting in an absolute 311 mGy reduction in reference point air kerma. The DAP also decreases from 166.34 to $12.14\;\mu {\mathrm{Gym}}^{2}$, representing a total reduction of an absolute $154.2\;\mu {\mathrm{Gym}}^{2}$ reduction in DAP. Importantly, with the AERC no longer focusing on the coil, Fig. 13(c) demonstrates clear visualization of the surrounding regions.

#### 5.3.3 Results: experiment 3

Table 6 outlines the outcomes from experiment 3, conducted to assess the time required by clinical experts for manual collimation. It was performed with two different detector sizes 30×40  cm and 21×21  cm. In neurosurgeries, a standard detector size of 30×40  cm is typically used. However, due to limitations in availability within our laboratory, we conducted tests using two different sizes. The mean time across all experts for manual collimation, regardless of the detector size, stands at 29.2 s. Thus the average time of 29.2 s serves as the lower limit for manual collimation in these cases.

**Table 6:** Time taken for manual collimation by experts, over an average of 3 trials per expert on two detector sizes 30×40  cm and 21×21  cm.

Expert	Time (s)	Detector size
1	47.17	30 cm × 40 cm
2	50	30 cm × 40 cm
3	16.18	21 cm × 21 cm
4	20.02	21 cm × 21 cm
5	13.08	21 cm × 21 cm

By incorporating automatic coil detection and subsequent collimation, the inference time is reduced to ∼1 to 2 s. Collimating around the coil after this automated detection and localization process takes less time than the average duration of manual collimation.

## 6 Conclusion

From our experiments, we have established the superiority of the deep learning-based coil detection method over the classical blob detection approach. The application experiments further highlighted the benefits of employing the coil detection algorithm for automatic collimation. This resulted in a notable reduction in radiation exposure and a decrease in the collimation time. Deploying such deep learning models in the current Fluoroscopy suites would also be possible because the current Siemens X-ray angiography systems are equipped with GPUs for image processing. Despite the advantages, we can still improve the method using a more extensive dataset to make it robust against false positives and testing other object detection techniques.

We could also use our approach to understand the workflow of the coiling procedure, where we currently know the end of it by detecting the coil. As a next step, we are working on detecting the framing coil and the start of the coiling procedure.

## Data Availability

The data used in this experiment were analyzed retrospectively. The data were obtained from a contract made between Siemens and the Hospital which states that the data can be used for any experiments and publication of Siemens. There were no changes made with respect to the treatment of the patient and only the acquired images were used for the experiment. The acquired data are in the so-called raw format, which makes it impossible to obtain any patient information. Informed consent has been obtained from all the patients. The study was performed according to the regulations and guidelines of Siemens and was approved by the Siemens Quality Committee “AT-22-627 Deep learning-based embolization coil detection for Automatic Collimation in Interv X-ray.” The data are not publicly available but can be provided on special request.
